# Virulence profiles and innate immune responses against highly lethal, multidrug-resistant nosocomial isolates of *Acinetobacter baumannii* from a tertiary care hospital in Mexico

**DOI:** 10.1371/journal.pone.0182899

**Published:** 2017-08-10

**Authors:** Roberto Rosales-Reyes, Catalina Gayosso-Vázquez, José Luis Fernández-Vázquez, Ma Dolores Jarillo-Quijada, César Rivera-Benítez, José Ignacio Santos-Preciado, María Dolores Alcántar-Curiel

**Affiliations:** 1 Unidad de Investigación en Medicina Experimental, Facultad de Medicina, Universidad Nacional Autónoma de México. Ciudad de México, México; 2 Hospital General de México, Dr. Fernando Liceaga, Ciudad de México, México; Chang Gung University, TAIWAN

## Abstract

Virulence profiles and innate immune responses were studied in *Acinetobacter baumannii* from nosocomial infections collected over one year in a tertiary care hospital in Mexico. *A*. *baumannii* were identified by VITEK 2 System followed by susceptibility tests. Carbapenemase genes, active efflux mechanism to imipenem and meropenem and outer membrane proteins profile were analyzed to evaluate their role on the activity of carbapenem resistance. All isolates were genotyped by pulsed field gel electrophoresis. The ability to form biofilm was determined on a polystyrene surface. The resistance to complement was determined with a pooled human normal serum and TNFα release by infected macrophages was determined by ELISA. The 112 isolates from this study were associated with a 52% of mortality. All were resistance to β-lactams, fluoroquinolones, and trimethroprim-sulfamethoxal, 96 and 90% were resistant to meropenem and imipenem, respectively, but with high susceptibility to polymyxin B, colistin and tigecyclin. Isolates were classified in 11 different clones. Most isolates, 88% (99/112), were metallo-β-lactamases and carbapenemases producers, associated in 95% with the presence of *bla*_OXA-72_ gene. Only 4/99 and 1/99 of the carbapenem-resistant isolates were related to efflux mechanism to meropenem or imipenem resistance, respectively. The loss of expression of 22, 29, and/or 33-36-kDa proteins was detected in 8/11 of the clinical isolates with resistance to carbapenem. More than 96% (108/112) of the isolates were high producers of biofilms on biotic surfaces. Finally, all isolates showed variable resistance to normal human serum activity and were high inductors of TNFα release by macrophages. In summary, these results suggest that multidrug-resistant *A*. *baumannii* can persist in the hospital environment through its ability to form biofilms. The high mortality observed was due to their ability to survive normal human serum activity and capability to induce potent inflammatory immune response making this nosocomial pathogen a serious threat to hospitalized patients.

## Introduction

Over the last few years, infections involving species of Gram-negative non-enteric and multidrug-resistant bacteria have been increasing worldwide [1]. Among these bacteria, *Acinetobacter baumannii* has emerged as the most important opportunistic pathogen involved in serious hospital infection outbreaks [2, 3]. These bacteria have been isolated from different environmental sources, including soil, water, food products, and medical devices as well from the skin of hospital staff. [4–12]. *A*. *baumannii* is a bacterium that has acquired diverse mechanisms of resistance to several antibiotic families, which has led to the emergence of important multidrug or pandrug resistant phenotypes [1]. Until recently, the carbapenems were the first antibiotics of choice in the treatment of nosocomial infections, including *A*. *baumannii* [13]. The emergence of resistance by *A baumannii* to carbapenems has been reported from different parts of the world [14]. The carbapenem resistance by *A*. *baumannii* is due to: a) the production of class B carbapenemases also known as metallo-β-lactamases (MBLs) and class D enzymes (also known as oxacillinases), b) genetic alterations of penicillin binding proteins, c) overexpression of efflux pumps belonging to the resistance-nodulation-cell division (RND) family and d) the loss of outer membrane proteins associated with the formation of pores [15]. The increased multidrug resistance and the persistence for long periods of time in hospitals make *A*. *baumannii* a serious threat to hospitalized patients. The capability of *A*. *baumannii* to form biofilm on biotic or abiotic surfaces partially explains the persistence of these bacteria in the hospital environment [16, 17].

To date, few virulence mechanisms have been described in *A*. *baumannii* [18]. The lipopolysaccharide (LPS) of *Acinetobacter* is a potent inducer of the inflammatory immune response through the stimulation of tumor necrosis factor alpha (TNFα) release by phagocytic cells via Toll-like receptor 4 (TLR4) signaling [19]. In addition, *A*. *baumannii* has the ability to resist the killing action of normal human serum (NHS) [20]. This serum resistance is attributed in part to LPS expression [20].

In the present study, we analyzed clinical nosocomial isolates of *A*. *baumannii* from a tertiary care hospital in Mexico City by their antimicrobial susceptibility profile, clonal relationships, ability to form biofilms on abiotic surfaces, by their capability to resist to human normal serum activity as well as their ability to induce TNFα release by phagocytic cells. Our results attempt to clarify the participation of antimicrobial resistance, virulence profiles and the innate immune response on the prevalence, endemicity and lethality of *A*. *baumannii* in critically ill patient in the hospital environment.

## Materials and methods

### Clinical isolates

The clinical isolates of *A*. *baumannii* used in this study were from patients with nosocomial infections during the study period, from January to December of 2014, at Hospital General de México Dr. Eduardo Liceaga, Mexico City, Mexico. The isolates were identified by the VITEK 2 System (bioMerieux, Marcy l'Etoile, France), confirmed by API20NE (bioMeriux®SA) [21] and by detecting the intrinsic carbapenemase *bla*_OXA-51-like_ gene. Only one isolate per patient episode was analyzed. All samples were collected as part of routine care, identified and handled anonymously. Nosocomial infections were defined according to the Centers for Disease Control and Prevention (CDC) criteria, and by Infectious Diseases Unit physicians [22].

### Antibiotic susceptibility testing

The isolate strains were tested for their susceptibilities to 17 antibiotics: gentamicin, tobramycin, cefepime, ceftriaxone, ampicillin/sulbactam, imipenem, meropenem, ciprofloxacin, levofloxacin, minocycline, tetracycline, trimethoprim/sulfamethoxazole, colistin, polymyxin B, and tigecycline. The minimum inhibitory concentrations (MICs) was determined using the VITEK 2 System and were confirmed using an agar dilution method, according to Clinical Laboratory Standards Institute (CLSI) guidelines [23, 24]. *Pseudomonas aeruginosa* ATCC 27853 and *E*. *coli* ATCC 25922 were used as positive and negative controls, respectively. In carbapenem-resistant isolates, MBLs were determined by EDTA disc synergy tests using meropenem and meropenem plus EDTA and the Hodge modified method [25].

### Detection of *bla*_OXA-51-like_, *bla*_IMP_, *bla*_VIM_, *bla*_OXA_ carbapenemase and IS*Aba*-1 genes

Chromosomal DNA was extracted from the isolates; 2-μL volume of this extract was used for PCR reactions. Amplification of *bla*_OXA**-**51-like_, *bla*_IMP_, *bla*_VIM_ and *bla*_OXA_ genes was performed using primers and conditions of amplification as previously reported [15, 26, 27]. The presence of the IS*Aba*1 promoter sequence and its association with carbapenemase genes was investigated by PCR using IS*A*ba-1 [28], IS*Aba*1 + *bla*_OXA-51-like_ and IS*Aba*1 + *bla*_OXA_ primers [15]. Amplified products were subjected to nucleotide sequencing at the Instituto de Biotecnología, Universidad Nacional Autónoma de México.

### Pulsed-field gel electrophoresis (PFGE)

To determine the spread of *A*. *baumannii* within the hospital setting, all isolates were genotyped using Pulsed-Field Gel Electrophoresis (PFGE). Bacterial genomic DNAs were prepared as previously described [15], digested with *Apa*I (New England Biolabs, Beverly, MA) and subjected to PFGE analysis using a Gene Path system (BioRad®). Tenover criteria and Dice coefficient were used to determine similar profiles between isolates, a correlation > 85% were considered to have the same pulsotype (clones) [29].

### Detection of the efflux pumps phenotype

In order to determine the role of the efflux mechanisms, all carbapenem-intermediate or-resistant isolates were selected to identify the inhibitory effect of the efflux inhibitor carbonil cyanide 3-chlorophenylhydrazone (CCCP) (Sigma, St. Louis, MO) on the change of meropenem or imipenem susceptibility had on the isolates. Briefly, Müeller-Hinton agar plates with meropenem or imipenem double serial dilution in the presence or absence of 25, 50, and 100 mg/L CCCP inhibitor were used [30]. Positive contribution of an efflux pump was defined as a four-fold decrease in the MIC of antibiotics when efflux pump inhibitor was added to the agar plates [30].

### Outer membrane proteins (OMPs) profile

The *A*. *baumannii* OMPs expression from one representative isolate of each carbapenem-resistant clone identified in this study were used to support the contribution of the porin profile modification in the carbapenem-resistance. As a control we used a carbapenem-susceptible *A*. *baumannii*-176 clinical isolate [15]. The OMPs were prepared as previously described [31]. Twenty μg of extracted proteins of each sample were resolved in one-dimensional sodium dodecyl sulfate-polyacrylamide gel electrophoresis (SDS-PAGE). Silver staining (Silver Stain Plus BioRad®) was used to visualize the protein bands.

### Biofilm production

The biofilm production on an abiotic surface was quantified as previously described [32]. Briefly, 5 ml overnight cultures grown at 37^*°*^C were diluted to OD_*600*_ 0.003 in Luria Bertani (LB) media and triplicate 500 μl aliquots were dispensed into polystyrene tubes. Following 24 h of static incubation at 37^*°*^C, the medium was removed and the tubes were washed gently once with deionized water. Adherent bacteria were stained with 1% (w/v) crystal violet and washed three times with deionized water. The bound crystal violet was dissolved in 1 ml of 100% methanol and quantified by measuring OD_*540*_ nm.

### Serum resistance assay

The assay was conducted as previously reported [33] with few modifications. Briefly, *A*. *baumannii* isolates were grown in Müller Hinton (MH) broth to the mid-log phase. We adjusted the inoculum to 1x10^*7*^ bacteria with 40% [in phosphate buffered saline (PBS)] either pooled normal human serum (NHS) (Cedarlane Laboratories Limited, Ontario, Canada) or with heat-inactivated human serum (HIS), the samples were incubated for 3 h at 37^*°*^C without shaking. After the incubation, the number of surviving bacteria in each sample was determined by a serial dilution and plating in MH agar at 37^*°*^C for 18 h. The serum bactericidal effect was calculated with the following formula: (CFUs-NHS / CFUs-HIS)*100. NHS and HIS were assessed using *Salmonella typhi* 9:12:Vid as a sensitive strains to determine the complement pathway activated by these bacteria [34]. All experiments were performed in triplicate and results were expressed as percent of survival.

### TNFα release assay

RAW 264.7 (ATCC TIB-71) macrophages were grown in 24-well plates at 2.5x10^5^ cells per well and incubated during 24 h. Overnight bacterial cultures were grown in MH broth at 37°C with shaking. Bacterial cultures were washed twice with RPMI plus 10% fetal bovine serum (FBS) and used to infect macrophages monolayers at an MOI of 100 as described [35]. Briefly, to synchronize the infection, plates were centrifuged for 1 min at 1400 rpm and incubated for 3 h at 37°C under 5% carbon dioxide. After infection, the supernatants were removed and centrifuged at 14000 rpm to remove bacteria. The supernatants were evaluated for the TNFα-release by ELISA (R&D systems) as described [36].

### Statistical analysis

The data represents the mean of the standard deviation (SD) and were analyzed by one-way ANOVA followed by a post hoc Tukey’s comparison. In some cases, data with normal distribution were analyzed using Student t test. The p values, 0.05 was considered significant. The data were analyzed using GraphPad Prism 6 software.

## Results

### Clinical isolate data

A total of 112 *A*. *baumannii* isolates causing nosocomial infections were identified. The mean age of patients was 47.56 years (ranging of 0.4 to 83), 66.07% of them were male. The mortality rate in this study was 51.78% (58/112); the majority of the deaths 27/58 occurred in the Pulmonary Medicine Ward (manuscript in preparation). The *A*. *baumannii* clinical isolates were obtained from different sources: 50% (56/112) of them were from respiratory sources, 23.2% (26/112) from wound secretions, 14.2% (16/112) from blood culture, 7.1% (8/112) from urine, 3.6% (4/112) from cerebral spinal fluid, 0.9% (1/112) from vascular catheter and 0.9% (1/112) from eye drainage. The most frequent site of isolation 38% (43/112) was the Pulmonary Medicine Ward, with 13% (15/112) the Intensive Care Unit, 9% (10/112) the Infectious Diseases Unit and 7% (8/112) the Surgery Ward. The remaining isolation sites 32.1% (36/112) were from various medical subspecialties and surgical wards.

### Antibiotic susceptibility

All *A*. *baumannii* isolates were confirmed by amplification of *bla*_OXA-51-like_ gene. The isolates were 100% resistant to β-lactams, fluoroquinolones and trimethoprim-sulfamethoxazole, 96.4% with resistance to meropenem and 89.2% to imipenem (Table 1). All isolates were susceptible to colistin and tigeciclyne and only the 2.7% of them were resistant to polymyxin B (Table 1). MBL activity was found in 88.4% (99/112) carbapenem-resistant isolates (Table 2). By PCR and sequencing we detected in 95% (94/99) of the isolates the *bla*_OXA-72_ gene (GenBank accession number: JX968505.1). None of the isolates were carrying either *bla*_VIM_ or *bla*_IMP_ genes (Table 2). Insertion sequences IS*Aba*-1 were detected in all of the isolates, however, *bla*_OXA-51-like_ or *bla*_OXA-72_ genes were not adjacent to the IS*Aba*-1.

**Table 1:** Antibiotic susceptibility for 112 nosocomial isolates of *Acinetobacter baumannii*.

Antibiotic family	Antibiotic	Breakpoints (CLSI/2015)			MIC_50_ (μg/mL)	MIC_90_ (μg/mL)	Susceptible (%)	Intermediate (%)	Resistant (%)
		S	I	R					
Aminoglycosides	Gentamicin	≤4	8	≥16	8	16	27.7	60.7	11.6
	Tobramycin	≤4	8	≥16	8	8	56.2	42	1.8
Cephems	Cefepime	≤8	16	≥32	≥64	≥64	0.9	0.0	99.1
	Ceftriaxone	≤8	16–32	≥64	≥64	≥64	0.0	0.0	100.0
β-lactam/beta-lactamase inhibitor combinations	Ampicillin/ sulbactam	≤8/4	16/8	≥32/16	16	32	38.4	46.4	15.2
Carbapenems^a^	Imipenem	≤2	4	≥8	16	32	1.8	9.0	89.2
	Meropenem	≤2	4	≥8	16	32	0.9	2.7	96.4
Fluoroquinolones	Ciprofloxacin	≤1	2	≥4	≥4	≥4	0.0	0.0	100.0
	Levofloxacin	≤2	4	≥8	≥8	≥8	0.0	0.9	99.1
Tetracyclines	Minocycline	≤4	8	≥16	≤1	4	90.2	6.2	3.6
	Tetracycline	≤4	8	≥16	4	16	71.4	14.3	14.3
Folate pathway inhibitors	Trimethoprim/sulfamethoxazole	≤2/38	-	≥4/76	≥320	≥320	0.0	0.0	100.0
Lipopetides	Colistin	≤2	-	≥4	≤0.5	≤0.5	100.0	0.0	0.0
	Polimyxin B	≤2	-	≥4	1	2	97.3	0.0	2.7
Glycylcycline	Tigecycline	≤2	4	≥8	2	2	92.2	7.1	0.0

The susceptibility profile was determined with the Vitek 2 automated system.

^a^The susceptibility to carbapenems profile was determined by doubling dilutions in agar (CLSI/2015).

**Table 2:** *Acinetobacter baumannii* clones and mechanisms associated with carbapenem resistance.

Clone *n* = 112	*bla*_OXA-51_ gene*n* = 112	IS*Aba1*gene *n* = 112	IS*Aba1*-*bla*_OXA-51_*n* = 0	*bla*_OXA-72_ gene*n* = 94	IS*Aba1*-*bla*_OXA-72_*n* = 0	MBLs phenotype*n* = 99	*bla*_VIM_ gene*n* = 0	*bla*_IMP_ gene*n* = 0	Activity efflux pump to IPM*n* = 1	Activity efflux pump to MEM*n* = 4
A*n* = 2	2/2	2/2	0/2	0/0	0/0	0/2	0/0	0/0	0/2	0/2
B*n* = 34	34/34	34/34	0/34	32/34	0/32	32/34	0/32	0/32	0/32	0/32
*Cn* = 23	23/23	23/23	0/23	21/23	0/21	22/23	0/22	0/22	0/22	0/22
D*n* = 4	4/4	4/4	0/4	4/4	0/4	4/4	0/4	0/4	0/4	0/4
*En* = 1	1/1	1/1	0/1	1/1	0/1	1/1	0/1	0/1	0/1	0/1
F*n* = 15	15/15	15/15	0/15	13/15	0/13	15/15	0/15	0/15	1/15	1/15
G*n* = 1	1/1	1/1	0/1	1/1	0/1	1/1	0/1	0/1	0/1	0/1
H*n* = 10	10/10	10/10	0/10	0/10	0/0	2/10	0/2	0/2	0/2	1/2
I*n* = 12	12/12	12/12	0/12	12/12	0/12	12/12	0/12	0/12	0/12	2/12
J*n* = 1	1/1	1/1	0/1	1/1	0/1	1/1	0/1	0/1	0/1	0/1
K*n* = 9	9/9	9/9	0/9	9/9	0/9	9/9	0/9	0/9	0/9	0/9

### Genotyping of *A*. *baumannii* isolates

**Fig 1:**
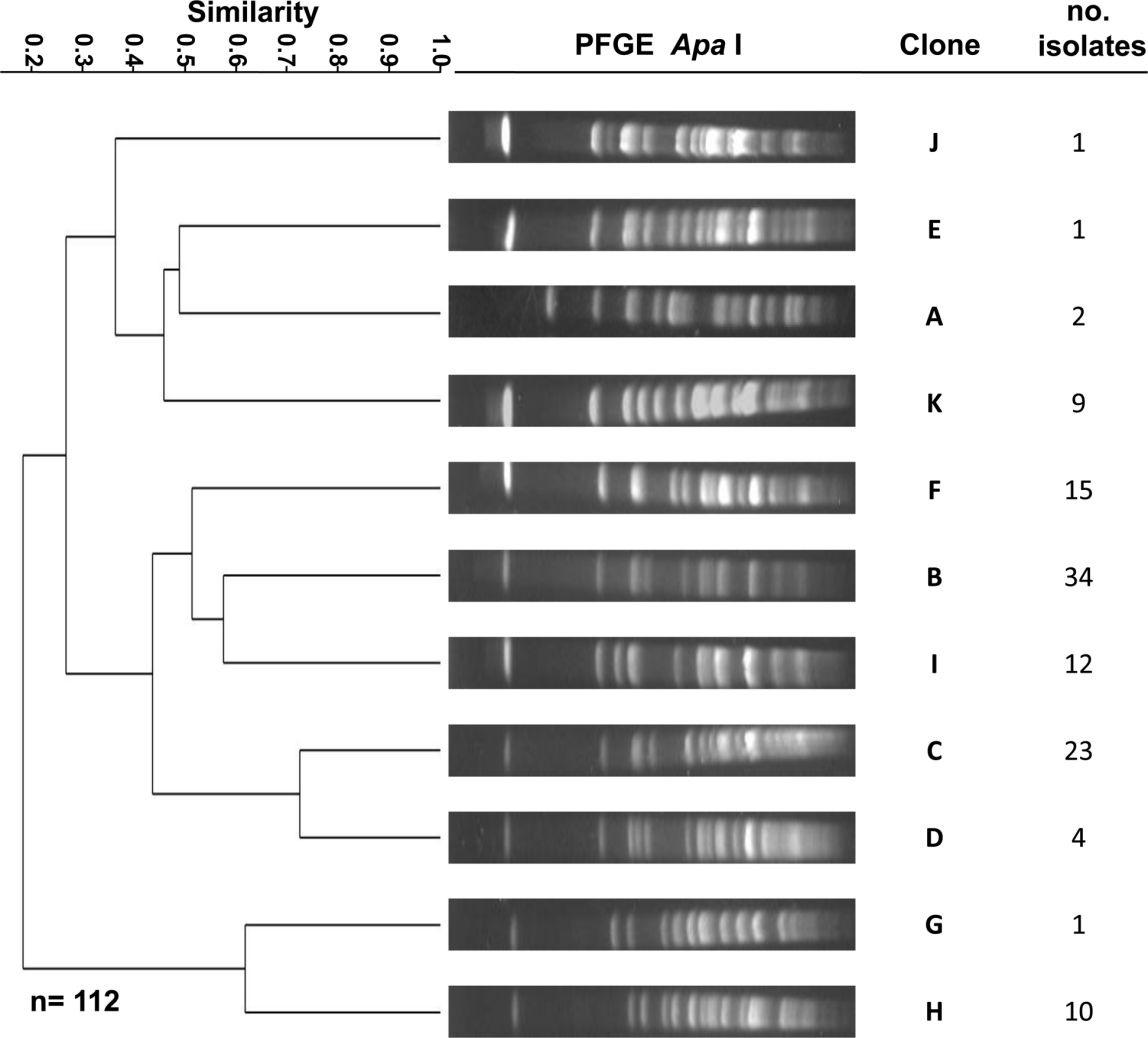
Dendrogram constructed from PFGE patterns of one representative isolate for each *Acinetobacter baumannii* clone. Clone B contained most of the isolates.

**Fig 2:**
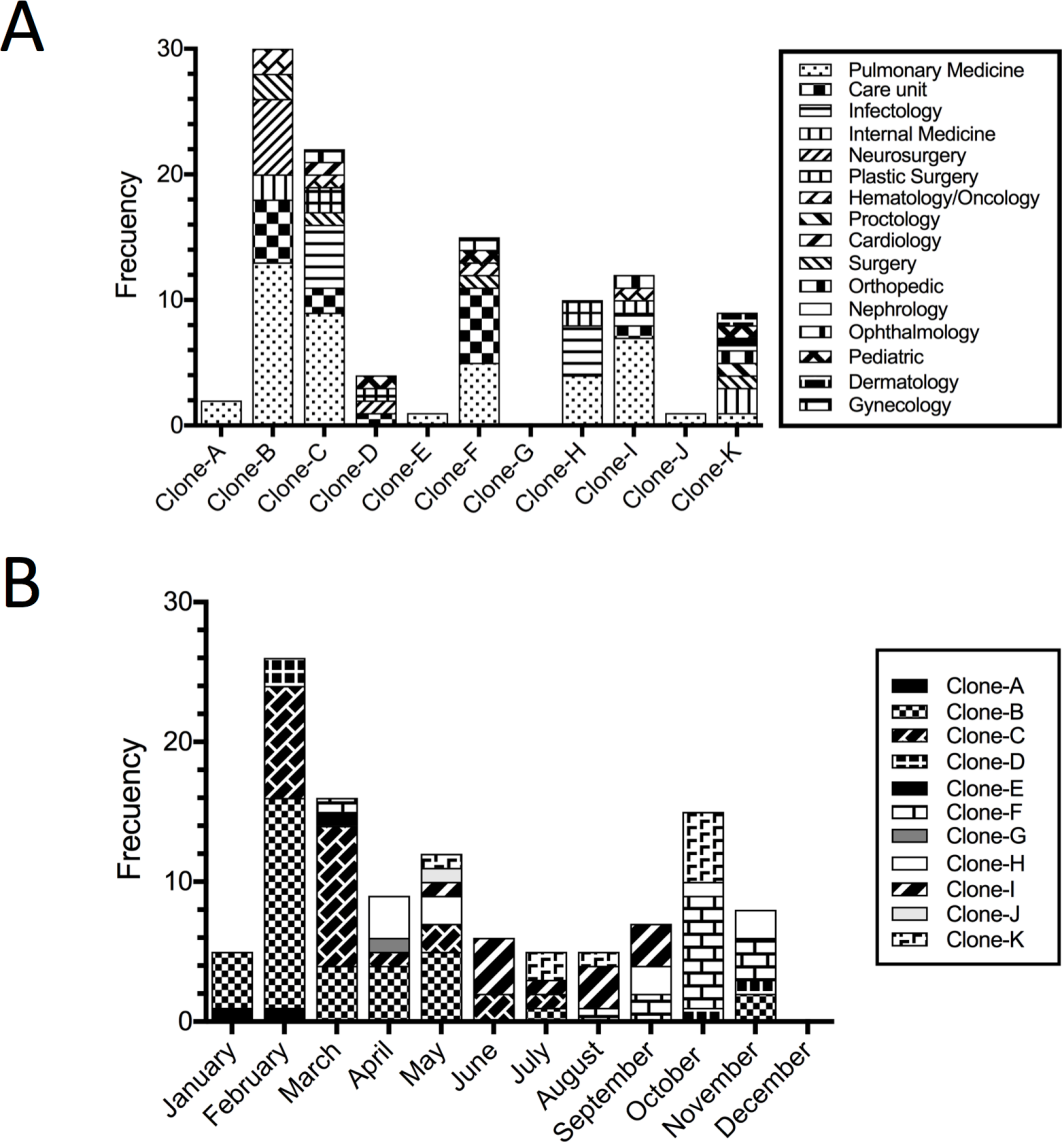
Frequency analysis of *A*. *baumannii* clones during 2014. (A) Frequency of each clone isolated by ward. (B) Frequency of each clone by month of isolation.

PFGE fingerprinting analysis revealed 11 clones in the 112 *A*. *baumannii* clinical isolates studied that were classified by assigning them letters from A to K (Fig 1). Nine clones were endemic in the Pulmonary Medicine Ward (Fig 2A). Clone B was the most frequently detected in 30% (34/112) of the clinical isolates (Fig 2B) and was responsible for an outbreak between February and March (Fig 2A and 2B). Clone B was also detected 7 times in the Pulmonary Medicine Ward and 4 times in the Intensive Care Unit (Fig 2A). Clone C was the second most frequently identified with 20.5% (23/112) of the isolates. This clone was detected between February-July and was also associated with the outbreak of February-March (Fig 2B). This clone was identified 7 times in the Pulmonary Medicine Ward and 5 times in the infectious diseases unit (Fig 2A). The third most frequent clone was the clone F with 15 isolates 13%. Members of this clone were identified at the end of the study (Fig 2B).

### Efflux pump phenotype

Results indicated that in the minority of the carbapenem-intermediate or -resistant isolates, efflux pump expression was not related to the carbapenem resistant phenotype (Table 2). Only 4/112 isolates exhibited a four-fold or greater decrease in MICs to meropenem. These isolates belonged to three different clones, two were from clone I, one from clone D and one from clone H. Only one isolate (0.89%) from clone F exhibited efflux pump activity to imipenem (Table 2). None of the isolates resistant to imipenem and meropenem exhibited an efflux pump to both carbapenems.

### OMPs profiles

The OMPs profiles of the one member or each clone carbapenem-resistant were obtained. In agreement with previous studies, our results showed that 72.7% (8/11) of the OMPs profiles had the absence of one, two or three of proteins of 22, 29, and/or 33-36-kDa, compared to the OMPs profile of the carbapenem-susceptible *A*. *baumannii* isolate (Table 3).

**Table 3:** Loss of porin expression in *A*. *baumannii* associated with carbapenem resistance.

Isolate	Clone	IMP MIC(μg/mL)		MEM MIC(μg/mL)		Loss of OMPs associated with carbapenem resistance
001	A	32	R	16	R	None
005	B	16	R	16	R	None
014	C	32	R	16	R	22kDa, 29kDa, 33-36kDa
013	D	128	R	16	R	22kDa, 29kDa, 33-36kDa
057	E	32	R	16	R	None
117	F	16	R	8	R	22kDa
068	G	128	R	128	R	29kDa, 33-36kDa
136	H	4	I	16	R	29kDa, 33-36kDa
101	I	32	R	8	R	29kDa, 33-36kDa
093	J	64	R	16	R	22kDa, 29kDa
131	K	16	R	16	R	22kDa, 29kDa

### Biofilm production

**Fig 3:**
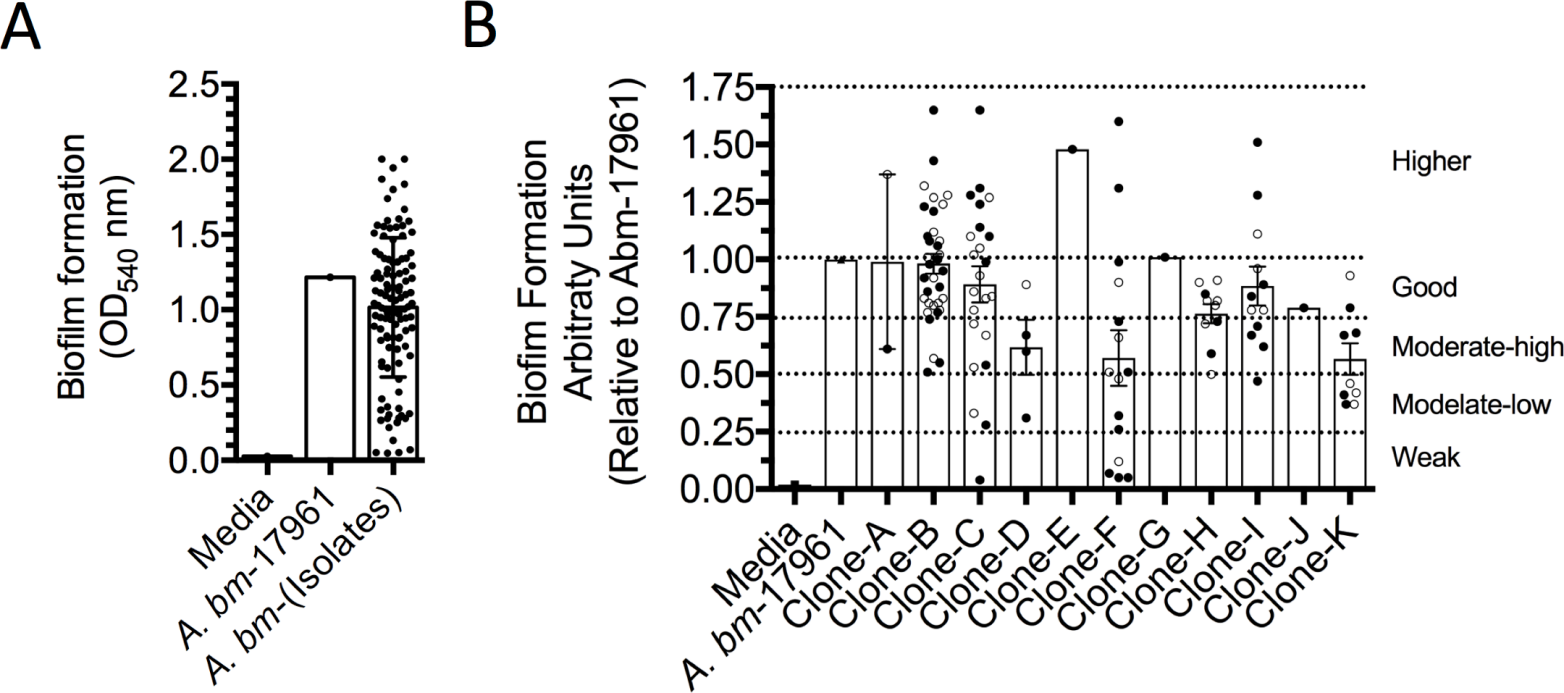
Ability of the *A*. *baumannii* clones to produce biofilm. All isolates were assessed for their ability to produce biofilm on a polystyrene surface. (A) We show the ability of each isolate to form biofilm. (B) We present the biofilm formation by the members of each clone. Open circle indicate patients that improved and closed circles correspond to patients that died. *A*. *baumannii* ATCC 17961 was used as the control. The dotted lines indicate weak, moderate-low, moderate-high, good and high production of biofilm. Each point corresponds to the average of two independent experiments by duplicate. Each column indicates the standard deviation.

The ability of each *A*. *baumannii* isolate to form biofilm is summarized in Fig 3A. The OD_450_ values for the reference strain (ATCC-17961) and negative control (MH broth) were 1.215 and 0.026 respectively. The OD_450_ for the reference strain (ATCC-17961) was defined as 1.0, the values presented in the Fig 3B represent the biofilm formation of every clone in relation to the biofilm formed by *A*. *baumannii*-ATCC-17961. The results show that the 96.4% (108/112) of the clinical isolates form biofilm on abiotic surface and only 4/112 (3.6%) produce weak biofilm (less than 5% amount relative to ATCC-17961) (Fig 3A). In the Fig 3B, we present an analysis of all isolates classified by clone. The results indicate that 13/112 were weak biofilm producers (represents the 0–25% of the biofilm produced by ATCC-17961); 7/112 produced moderate-low biofilm (25–50% of the biofilm formed by ATCC-17961). In contrast, 17/112 (15.18%) were moderate-high biofilm producers (50–75% of the biofilm formed by ATCC-17961). Of note, 33.04% (37/112) were good biofilm producers (75–100% of the biofilm produced by ATCC-17961), and 36/112 (32.14%) were high biofilm producers (more than the biofilm formed by ATCC-17961) (Fig 3B**).**

### Serum resistance assay

**Fig 4:**
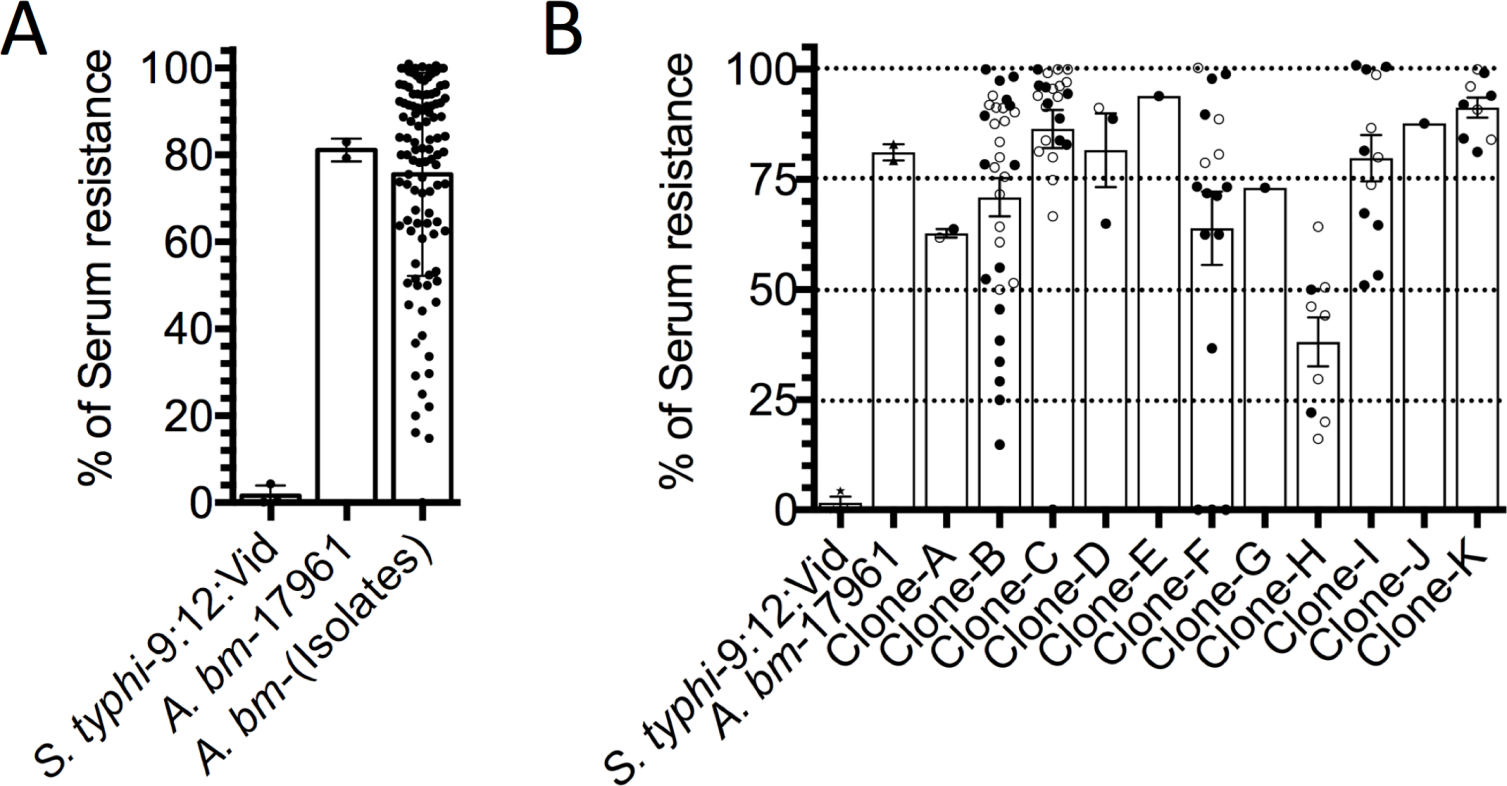
Analysis of serum resistance activity by *A*. *baumannii* clones. Each *A*. *baumannii* isolate was assessed by its ability to survive in normal human serum (NHS). (A) We show the percentage of *A*. *baumannii* isolates to survive in presence of 40% of NHS. (B) We show the ability of the members of each clone to survive at 40% of NHS. The dotted lines indicate the survival rate (0–25, 26–50, 51–75 and 76–100%) in presence of 40% of NHS. Open circle indicate patients that improved and every closed circle corresponded to a patient that died. Each point corresponds to the average of two independent experiments by duplicate. Each column indicates the SD.

Evidence from the literature indicates that clinical isolates of *A*. *baumannii* show a differential capacity to survive NHS activity [33, 37]. In this study, we quantified the capacity of all clinical isolates to survive to the NHS, as a control we used NHS-heat inactivated (HI). The percent survival of each clinical isolate after incubation for 3 h in 40% of NHS is presented in Fig 4A. Under these conditions, *S*. *typhi*, the control strain is highly serum-sensitive. The results in Fig 4B show that 6/112 (5.35%) of the isolates presented a survival rate of 1–25% in NHS, 11/112 (9.82%) a survival rate of 26–50% in NHS, 26/112 (23.21%) a survival rate of 51–75% and 69/112 (61.60%) a survival rate of 76–100% in the presence of NHS. In summary, the clinical isolates showed differential susceptibility/resistance to NHS, however, the majority of the clinical isolates were resistant.

#### Quantitation of TNFα release by macrophages

**Fig 5:**
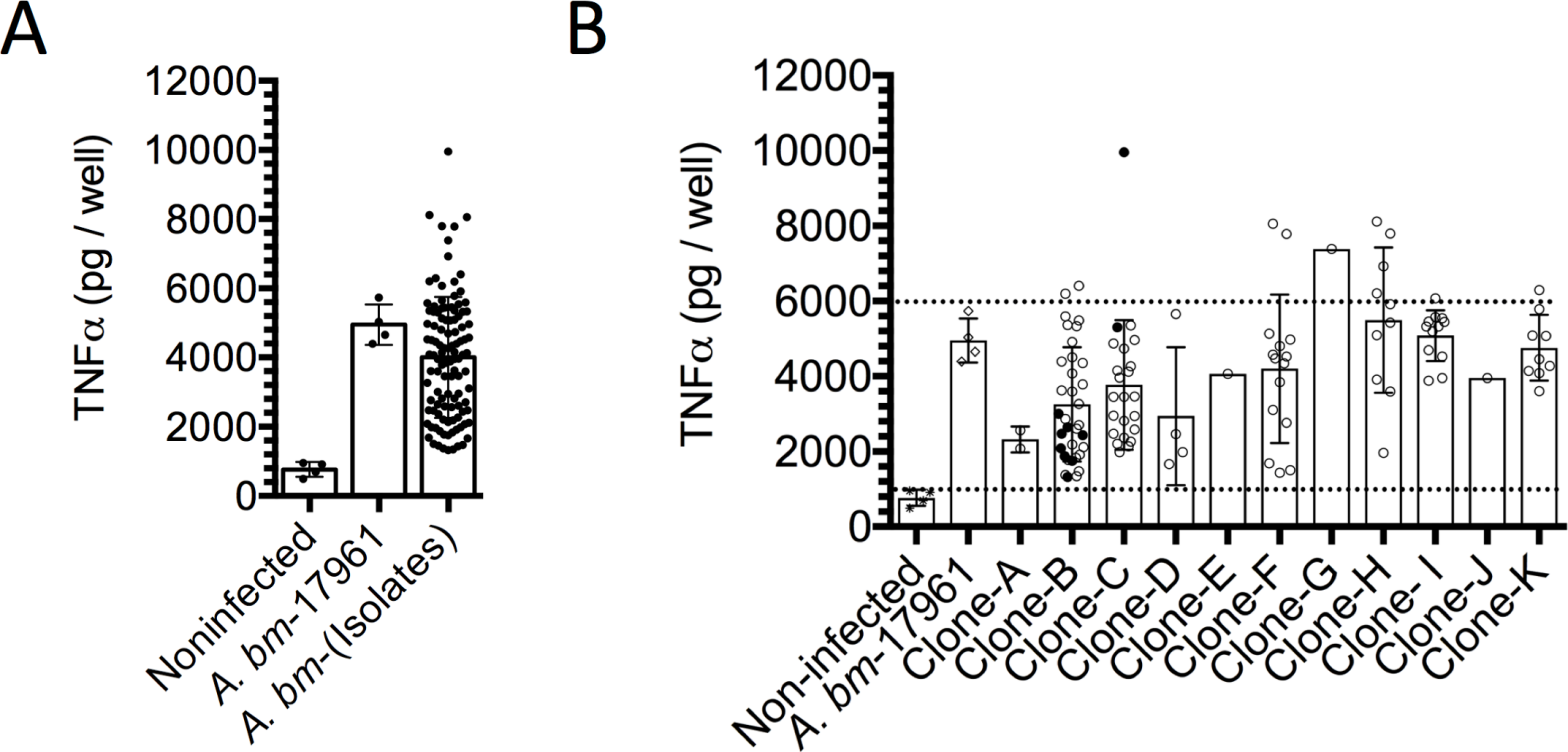
Ability of the *A*. *baumannii* clones to produce TNFα. Each *A*. *baumannii* isolate was assessed by its ability to induce the release of TNFα by macrophages. (A) We show the production of TNFα by each *A*. *baumannii* isolate. (B) We show the production of TNFα by the members of each clone. The dotted lines indicate 1000 and 6000 pg/ml of TNFα. Open circle indicate patients that improved and closed circles corresponded to patients that died. Each point corresponds to the average of two independent experiments by duplicate. Each column indicates the standard deviation.

During the interaction between *A*. *baumannii* with phagocytic cells, the TLR4 recognizes the LPS of *A*. *baumannii* with the consequent release of TNFα [19]. In order to determine the ability of *A*. *baumannii* isolates to induce TNFα release by macrophages, we infected macrophages for 3 h and then quantified TNFα release. The results presented in the Fig 5 show that all *A*. *baumannii* isolates were able to induce more than 1500 pg/ml of TNFα and only 9.8% (11/112) isolates were higher inducers of TNF-α release (more than 6000 pg/ml). These results indicate that *A*. *baumannii* can be a potent inductor or inflammatory immune response.

## Discussion

During the last two decades *A*. *baumannii* has emerged as an important nosocomial pathogen, especially in hospitalized patients requiring intensive care [38]. One of the main factors that characterize clinical *A*. *baumannii* isolates is the abundance of plasmids, transposons and frequent genetic exchanges that confer multidrug-resistance, limiting therapeutic options which often leads to high mortality rates in hospitalized patients [1, 39].

This work was carried out in one of the major tertiary hospitals in Mexico City. The hospital has 1,245 beds, with an average of 45,000 discharges per year. During 2014, 112 clinical *A*. *baumannii* isolates from nosocomial infections were collected. The isolates exhibited high resistance to multiple antibiotics including 88% to carbapenems. These results are very similar to those we reported previously in a tertiary hospital in western Mexico [15] as well as reports from others countries [40–42].

Carbapenem resistance in the isolates studied was associated mainly with MBLs 88.4% and *bla*_OXA-72_ (83.9%) production. Since 2012, *bla*_OXA-72_ has been reported in Asia and currently in different parts of the world, including western Mexico [15]. Although all isolates carried *bla*OXA-51, and a high proportion presented *bla*_OXA-72_, the high expression of these two genes was not colinear with the IS*Aba*1, similar to what has been reported previously [43]. In this study, *bla*_VIM-1_ or *bla*_IMP_ genes were not detected in MBLs producing *A*. *baumannii* isolates, indicating that these isolates carried other type of MBLs, not tested.

Regarding other mechanisms of carbapenem resistance, we detected a lesser proportion of efflux pump expression associated with meropenem (3.57%) or imipenem (0.89%) resistance; these results differ from previous studies which showed drug export by efflux pump reduced meropenem susceptibility among the vast majority 65.7% of nosocomial *A*. *baumannii* isolates [30]. We noted a decrease in the number of isolates expressing this mechanism when compared with the nosocomial *A*. *baumannii* isolates studied in previous work [15]. Regarding the analysis of OPMs profiles, we observed that a high number of isolates 72.1% (8/11) representing each of the carbapenem-resistant clones showed the absence of 1, 2 or 3 porins. This suggests that this mechanism contributes significantly to the resistance to carbapenems in *A*. *baumannii* isolates causing nosocomial infections in this hospital in central Mexico.

In this study, patients with *A*. *baumannii* infection showed higher mortality rate (52.8%) than those with *A*. *baumannii* infection into the Hospital Civil de Guadalajara, Mexico (28.2%) reported previously [32]. In other countries, the mortality rate due to bloodstream imipenem-resistant *A*. *baumannii* infections is ranges from 52.2 to 86.7% [39, 44]. We demonstrated that the spread of clones B and C were responsible for an outbreak during February and March 2014 and these clones persisted during the first six months of that year. Epidemiological analysis showed that both clones were the most frequent and together represented half of the isolates 50.8% (57/112) identified in this study. The vast majority of these clones 94.7% produced MBLs, which explains why at least half of *A*. *baumannii* infections producing MBLs are due to outbreaks. Interestingly, when we compared eleven PFGE profiles of the clones detected in this study with the PFGE profiles of the clones of *A*. *baumannii* recently reported in a previous study in hospital from western Mexico [15] we found that clone H, the fourth most frequent clone with 10 isolates, was identical to clone 22, the most frequent clone previously described [15]. These results demonstrate that this multidrug-resistant clone has spread at least between these two distant geographically area of Mexico. To better understand how this clone emerged or where it has spread, we compared the genotype of clone H with those obtained by enzymatic restriction enzymatic with *Apa*I and PFGE reported for clones disseminated internationally. We used the genotypes of European clones I-III representative strains of outbreaks from United Kingdom, Spain, the Netherlands, France, and Israel previously reported [45], and found no similarities between these and clone H. Our results show that genotyping is a methodology that allows us to monitor the geographic spread of epidemic pathogens, and understand their epidemiology.

The persistence of *A*. *baumannii* in the hospital environment is associated with their ability to form biofilms on diverse biomedically relevant surfaces [46]. A study developed by *Rodríguez-Baño et al*. [47] showed that the 63% of unrelated clinical isolates were able to produce biofilm on abiotic surfaces. In contrast, 92.2% of the members of a highly prevalent clone of *A*. *baumannii* in the Hospital Civil de Guadalajara, Mexico were able to produce biofilm [32]. Our results show that almost all unrelated isolates were biofilm producers. The high percentage of biofilm formation by unrelated clinical isolates could be due by the presence of one or more of the mechanism associated to biofilm formation including the presence of *bla*_*PER-1*_ gene [48, 49] or to the presence of genes that encode for the pili assembly system (*csuC*, *csuD and csuE*) [50], the autoinducer synthase gene (*abaI*) [51] or the OmpA expression [52].

*A*. *baumannii* exhibits several virulence factors [18], its ability to survive complement activity is one of them [20, 33, 37]. The OmpA expression contributes to complement resistance by direct binding to the complement regulator, factor H. Thus, *A*. *baumannii* inhibits complement activation [37]. Our results show that the clinical isolates of *A*. *baumannii* present variable resistance to human serum. This ability to survive complement activity could be due either OmpA [37] or LPS expression [20].

The *A*. *baumannii* interaction with phagocytic cells induces proinflammatory cytokine release. The LPS of *A*. *baumannii* is a potent stimulator of TLR-4 [19, 53]. Our results show, that all clinical isolates assessed were potent inductors of TNFα release. Previous studies have documented that TNFα release during *A*. *baumannii* infection contributes *in vivo* to lung cell death [54].

Based on our results, the high prevalence of nosocomial isolates of *A*. *baumannii* with high mortality rate observed in this study was due to multidrug-resistant phenotypes in conjunction of their ability to form biofilm on abiotic surfaces, their high resistance to normal human serum and potent capacity to induce macrophage TNFα release.
